# Effects of Wet Versus Dry Warming Techniques on the Hardness of Nasotracheal Tubes

**DOI:** 10.7759/cureus.116267

**Published:** 2026-09-15

**Authors:** Kenji Nakagawa, Yuta Mitobe, Kentaro Matsumiya, Yasuko Baba, Thang Toan Vu, Tetsuya Miyashita

**Affiliations:** 1 Anesthesiology, International University of Health and Welfare, Narita Hospital, Narita, JPN; 2 Perianesthesia Nursing, International University of Health and Welfare, Tokyo, JPN; 3 Research and Development, Mitobe Medicalcare Lab LLC, Nagasaki, JPN; 4 Anesthesiology, International University of Health and Welfare Mita Hospital, Tokyo, JPN; 5 Anesthesiology, Takagi Hospital, Ōkawa, JPN

**Keywords:** heating gloves, nasal intubation, nasotracheal tube, nosebleeds, tracheal tube

## Abstract

Background and aim

Nasotracheal intubation is widely used in oral and maxillofacial surgery but is frequently associated with epistaxis. To reduce mucosal trauma, pre-warming the tube with warm water is commonly used; however, time-dependent changes in tube hardness and limitations of warming methods remain concerns. This study aimed to evaluate the softening effect of dry warming with heated gloves compared with conventional warm-water-based warming.

Methods

Nasotracheal tubes (NT) were cut into 1 cm² sections and measured at room temperature. They were then warmed for 10 min in either 40°C warm water or with 55°C heated gloves. Hardness was measured at 5 s after warming ended and every 30 s for 5 min. Data were analyzed using repeated-measures ANOVA and Mann-Whitney U tests.

Results

The heated-glove group showed significantly greater softening than the warm-water group at 5 s after warming ended and at 30, 60, 120, and 150 s after warming (p < 0.05). By 240 s, both groups had recovered to approximately 90% of baseline hardness. Measurement reliability was good (intraclass correlation coefficient {ICC} = 0.78).

Discussion

Dry warming offers advantages over wet warming, including ease of preparation, reduced risk of burns or contamination, and consistent temperature control. Warming to 55°C was considered acceptable for short-term use with respect to tube material tolerance.

Conclusion

Dry warming with heated gloves provides greater early softening than warm water-based warming and may serve as a practical method for NT tube preparation. This study focused on physical properties; clinical outcomes such as pain, mucosal injury, or airway patency were not assessed. Further clinical studies are warranted.

## Introduction

Nasotracheal intubation (NI) is a key airway management technique widely used in oral, maxillofacial, and dental surgeries, particularly in cases where access to the oral cavity is critical or when cervical spine instability and post-operative fixation pose challenges to securing the airway [1,2]. Despite its clinical utility, epistaxis remains a common complication, with incidence rates of 50-80%, posing challenges to its routine use in surgical practice [1,3,4]. Research has demonstrated that pre-warming nasotracheal tubes (NTs) using warm water can mitigate mucosal trauma by enhancing tube flexibility [5]. However, the cumbersome nature of water-based warming methods, combined with safety risks such as burn injuries when temperatures exceed 45°C, has limited their widespread adoption in clinical settings.

Furthermore, although hardness can be measured using static or dynamic methods, previous studies have seldom examined the temporal dynamics of NT hardness following thermal softening [6]. This underscores the need for a practical, efficient warming method that avoids the constraints of wet-warming procedures while offering comparable or superior benefits. This study aimed to investigate the softening effects of a dry-warming method using electrically heated gloves in comparison with the conventional wet-warming method using warm water. Wet thermal softening has demonstrated efficacy in reducing complications such as post-operative sore throat and hoarseness [6]. Additionally, strategies aimed at minimizing epistaxis during NI have been explored extensively [7]. However, most existing research has focused on post-operative outcomes, with limited attention given to temporal changes in tube hardness induced by different warming techniques. By addressing this gap, the present study seeks to provide evidence for optimizing NT preparation methods, potentially improving patient outcomes during NI procedures.

## Materials and methods

Nasotracheal tube segments were prepared as 1-cm specimens and allocated to either the wet-warming group or the dry-warming group. In the wet-warming group, the specimens were immersed in water maintained at 40°C for 10 min, whereas in the dry-warming group, the specimens were warmed with electrically heated gloves set to 55°C for 10 min. Hardness was measured using a durometer before warming, at 5 s after warming ended, and every 30 s up to 300 s after warming. This protocol was applied uniformly to both groups to compare the time-dependent changes in tube hardness after each warming method. Figure 1 provides a schematic overview of the experimental protocol.

**Figure 1:**
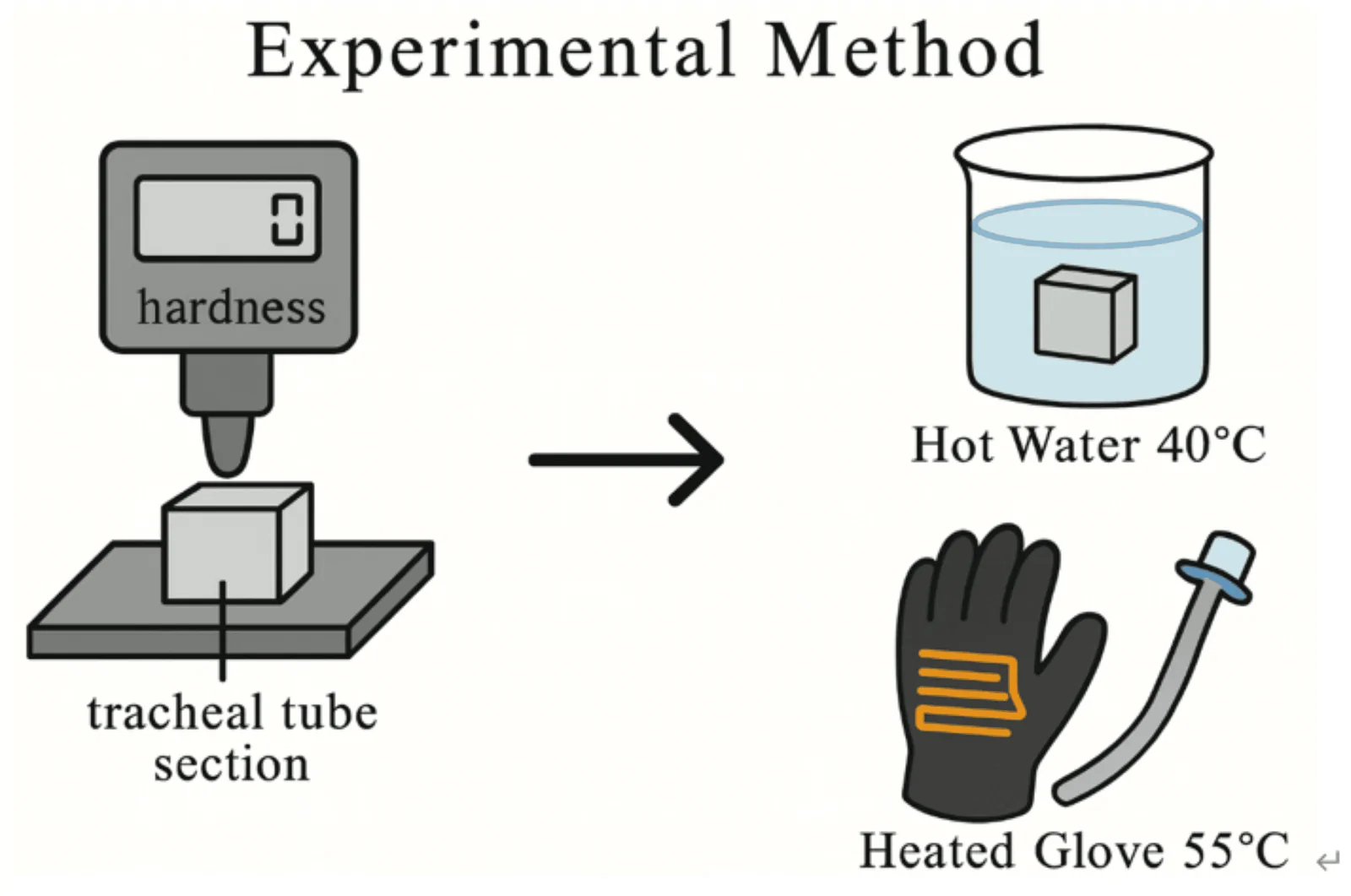
Schematic illustration of the experimental protocol. Nasotracheal tube (NT) segments of 1 cm were prepared and assigned to either the warm water group, in which segments were immersed in water maintained at 40°C for 10 min, or the heated glove group, in which segments were warmed using electrically heated gloves set at 55°C for 10 min, and hardness was measured using a durometer before warming, at 5 s after warming ended, and every 30 s for 5 min thereafter.

## Results

The changes in hardness over time are shown in Figure 2. Hardness decreased after warming in both groups and then gradually recovered over time. During the early post-warming period, the heated-glove group showed lower hardness values than the warm-water group, with significant between-group differences observed at the time points indicated by asterisks. Thereafter, the hardness values of the two groups gradually converged. From 180 s onward, no significant differences were observed between the groups, and by 240 s, both groups had recovered to approximately 90% of their initial hardness.

**Figure 2:**
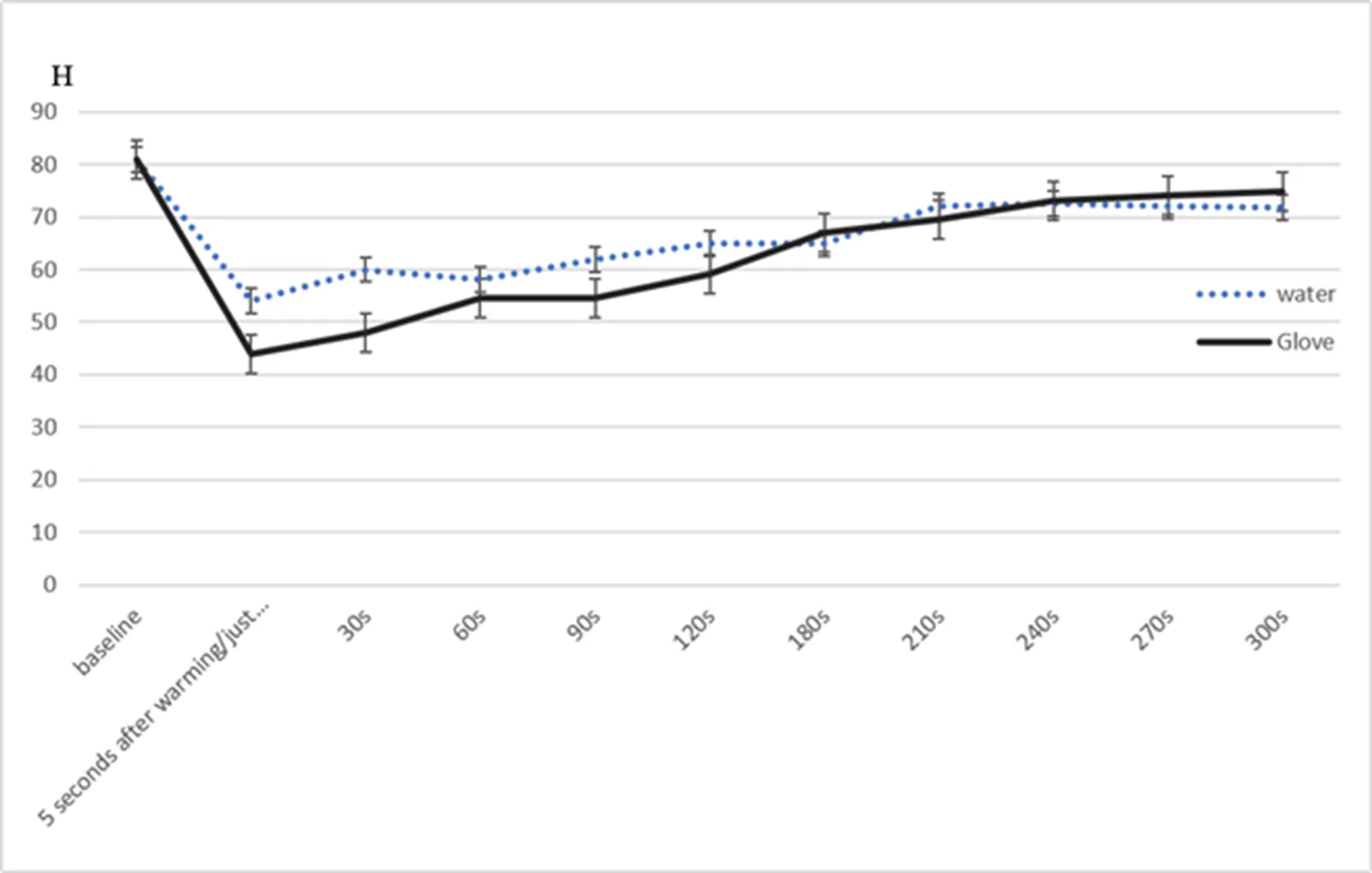
Time-dependent changes in nasotracheal tube hardness after warming. Hardness values of nasotracheal tube segments were measured before warming, at 5 s after warming ended, and every 30 s up to 300 s after warming. The warm water group was immersed in water at 40°C for 10 min, whereas the heated glove group was warmed using electrically heated gloves at 55°C for 10 min. Asterisks indicate statistically significant differences between the groups at each time point.

Table 1 shows the repeated-measures ANOVA for changes in nasotracheal tube hardness over time. The results of the repeated-measures analysis of variance (ANOVA) are shown in Table 1. This analysis examined the effects of warming method and time, and their interaction, on changes in nasotracheal tube hardness over time. The results revealed a significant main effect of the warming method (DEVICE), F (1, 8) = 16.73, p < 0.01. The main effect of time (TIME) was also statistically significant, F (10, 80) = 141.04, p < 0.01. Furthermore, a significant interaction between warming method and time (DEVICE × TIME) was observed, F (10, 80) = 9.54, p < 0.01. These findings indicate that changes in nasotracheal tube hardness differed significantly according to the warming method and measurement time.

**Table 1:** Repeated measures ANOVA for temporal changes in hardness. Heating method indicates the warming method, time indicates the measurement time point, and heating method × time indicates the interaction between warming method and time. Data were obtained from five samples per group (n = 5 per group).

Variables	df	F	p-Value
Heating method	1	16.73	<0.01
Time	10	141.04	<0.01
Heating method × time	10	9.54	<0.01

Table 2 presents between-group comparisons of nasotracheal tube hardness over time using the Mann-Whitney U test. The hardness values of the warm-water and heated-glove groups were compared at each time point using the Mann-Whitney U test. The results showed that at 5 s after warming ended and at 30, 60, 120, and 150 s, the hardness values in the heated-glove group were significantly lower than those in the warm-water group (p < 0.05), indicating more pronounced softening. From 180 s onward, the hardness values in the heated-glove group gradually recovered, and no significant differences were observed between the groups at 180 s or later. By 240 s, both groups had recovered to approximately 90% of their initial values.

**Table 2:** Between-group comparisons of nasotracheal tube hardness over time. Values are presented as median (interquartile range). Between-group comparisons at each time point were performed using the Mann-Whitney U test. IQR: interquartile range

Time point (n)	Warm water group (median {IQR})	Heated glove group (median {IQR})	p-Value
Baseline	81.0 (79.0, 83.0)	81.0 (79.0, 83.0)	1
5s	54.0 (53.0, 55.0)	44.0 (44.0, 45.0)	0.009
30s	60.0 (58.50, 62.0)	48.0 (48.0, 50.0)	0.009
60s	58.0 (57.50, 58.50)	54.50 (54.0, 55.0)	0.046
90s	62.0 (60.0, 63.0)	58.0 (57.0, 58.0)	0.075
120s	65.0 (65.0, 67.0)	59.0 (58.50, 59.50)	0.047
150s	67.50 (65.0, 68.0)	61.50 (60.50, 62.0)	0.012
180s	65.0 (63.0, 66.0)	67.0 (67.0, 69.0)	0.116
210s	72.0 (70.0, 72.50)	69.50 (67.50, 70.0)	0.209
240s	72.50 (70.0, 73.0)	73.0 (68.50, 74.0)	1
270s	72.0 (71.0, 73.0)	74.0 (71.50, 78.0)	0.295
300s	71.80 (69.0, 74.0)	74.70 (70.50, 79.0)	0.178

Reliability of the measurement method

This study evaluated the inter-rater reliability of the measurements using the intraclass correlation coefficient (ICC). A two-way random-effects model for absolute agreement based on single measurements was used (ICC {2,1}). The ICC was 0.78, indicating good inter-rater reliability. The F-test for the null hypothesis that the ICC equals zero was statistically significant, F (9, 6.39) = 10.90, p = 0.00342. The 95% confidence interval (CI) for the ICC ranged from 0.281 to 0.942. Although the lower bound of the CI was relatively low, the point estimate of the ICC fell within the range generally considered to indicate good reliability. These findings suggest that the measurement method demonstrated acceptable inter-rater reliability.

## Discussion

Warm water-based wet warming has traditionally been the dominant method for NT warming, as it enhances tube flexibility and reduces nasal trauma and epistaxis during intubation [8]. However, wet warming faces challenges with temperature control, with warming above 40°C posing risks of burns for both patients and medical staff, limiting its clinical application.

In contrast, dry warming using electric gloves - originally designed for hand warming in cold environments - offers warming up to 55°C. Short-term use for approximately 10 min is considered biologically safe.

This study quantitatively evaluated hardness changes in tracheal tubes from completion of warming to 300 s after 10 min of warming in both groups. The results demonstrated that the heated-glove group showed significantly greater softening at 5 s after warming ended and at 30, 60, 120, and 150 s compared with the warm-water group (p < 0.05), indicating enhanced warming effects during the early post-warming period.

Tracheal tubes are typically sterilized using ethylene oxide gas within a low temperature range of 30-60°C. From a material heat-resistance perspective, warming at 40°C and 55°C is considered safe. This study adhered to prior research by setting a 40°C limit for wet warming with warm water to mitigate burn risks to patients and medical staff. For dry warming, electric gloves were set to their maximum specification of 55°C.

Prior studies focusing on warming with warm water or saline at temperatures between 40°C and 50°C highlighted challenges, including strict control over water temperature and duration, which affect reproducibility and responsiveness. Additionally, practical constraints, such as the need for temperature regulators, sterile techniques, equipment disinfection, and risks of spills or burns, complicate its application [9].

In contrast, dry warming with electric gloves offers consistent and reproducible results by simply enclosing the tube once the set temperature is reached. It simplifies equipment preparation, reduces moisture-related damage and infection risks, and supports rapid responses during anesthesia induction. These factors, combined with the ability to safely achieve higher temperatures, underscore its clinical advantages.

## Conclusions

This study demonstrated that dry warming using electrically heated gloves achieved greater initial softening of nasotracheal tubes than wet warming with warm water. Given their simplicity, dry handling, and practical applicability, electrically heated gloves may represent a useful alternative method for preparing nasotracheal tubes in clinical settings. Further clinical studies are needed to determine whether this pre-warming method contributes to improved patient outcomes, including reduced post-operative pain, mucosal injury, and airway-related complications during nasotracheal intubation.
